# Chromosomal abnormality of acute promyelocytic leukemia other than *PML*-*RARA*: a case report of acute promyelocytic leukemia with del(5q)

**DOI:** 10.1186/s12907-016-0038-4

**Published:** 2016-10-04

**Authors:** Osamu Imataki, Makiko Uemura

**Affiliations:** Division of Hematology and Stem Cell Transplantation, Department of Internal Medicine, Faculty of Medicine, Kagawa University, 1750-1 Ikenobe, Miki-cho, Kita-gun, Kagawa 761-0793 Japan

**Keywords:** Acute promyelocytic leukemia (APL), *PML-RARA*, All-trans retinoic acid (ATRA), Arsenic oxide (ATO), Clonal cytogenetic aberration (CCA), Case report

## Abstract

**Background:**

The recent study described a better outcome in acute promyelocytic leukemia patients treated with all-trans retinoic acid and arsenic oxide compared to those treated with all-trans retinoic acid combined with conventional chemotherapy. The pivotal study indicated that favorable-risk acute promyelocytic leukemia patients can be cured without any cytotoxic chemotherapy. Even high-risk patients are treatable with cytotoxic agents. Acute promyelocytic leukemia does not develop only by the dedifferentiation caused by PML-RARA. A determined oncogene other than PML-RARA which promotes cell proliferation would be required.

**Case presentation:**

We recently treated a 30-year-old Japanese female who achieved molecular remission with only the administration of all-trans retinoic acid. The patient’s leukemic clones concomitantly had a del(5q) aberrant chromosome with t(15;17) (q22;q12). The patient’s bone marrow cells indicated clonal evolution of the tumor cells expressing CD13dim, CD33+, CD117+, and lacking HLA-DR, CD34 and CD11b. A fluorescence *in situ* hybridization analysis detected *PML-RARA* fusion genes in the patient’s bone marrow specimens, leading to the diagnosis of acute promyelocytic leukemia.

**Conclusion:**

A del(5q) is one of the characteristic chromosomal abnormalities observed in myelodysplastic syndrome. On the other hand, up to 40 % of acute promyelocytic leukemia cases are known to harbor the addition of a clonal cytogenetic abnormality. However, such a case acute promyelocytic leukemia with del(5q) would be rare, rather than myelodysplastic syndrome, consequently obtaining t(15;17). Which cytogenetic abnormalities, acute promyelocytic leukemia or myelodysplastic syndrome, came first is informative to make a clinical decision for the initial therapy. In this case, we speculated the *PML-RARA* translocation is an original pathogenesis and thereafter additional cytogenetic abnormalities (del(5q) and -6) common in myelodysplastic syndrome. All-trans retinoic acid lead the patient into molecular remission. We propose that an assessment of additional cytogenetic abnormality in acute promyelocytic leukemia would contribute to the clinical decisions regarding whether to treat disease with all-trans retinoic acid and cytotoxic agents. It would be of interest to know the extent of cytogenetic abnormality in the patients regarding to mixed leukemia. One or more additional cytogenetic abnormalities other than *PML-RARA* could account for the biological malignant grade and prognostic index.

## Background

Up to 40 % of patients with acute promyelocytic leukemia (APL) have an additional chromosomal abnormality other than *PML-RARA* [1], recognized as secondary cytogenetic abnormalities; +8 is the most frequent (10 %–15 %). The WHO 2008 criteria for the diagnosis of myelodysplastic syndrome (MDS) note that dysplasia in one or more lineages is essential and required for the diagnosis of MDS, and that the observation of several clonal chromosomal abnormalities, although frequent, is merely a supportive finding [1]. The presence of recurring chromosomal abnormalities as the sole finding in the absence of morphological changes is not considered definitive evidence of MDS.

The recurring chromosomal abnormalities characterized in MDS include +8, −7 or del(7q) −5 or del(5q), del(20q), −Y and others. Recurrent genetic abnormalities including t(15;17)(q22;q12) are the basis for categorizing a case as exclusively acute myeloid leukemia (AML). This means that any APL cases confirmed by the detection of t(15;17)(q22;q12) with myelodysplastic changes should be diagnosed as belonging in the AML category. A precise diagnosis is thus sometimes difficult in APL cases with morphologic changes such as myelodysplasia or secondary cytogenetic abnormalities [2].

Indeed, in a large-scale observational study of primary MDS, the translocation of chromosomes 15 and 17 was not noted among the 31 AML transformed from MDS (MDS/AML) [3]. If an individual is diagnosed as having APL with MDS, a question arises as to which condition developed first, the APL or the MDS. Myelodysplastic episodes that precede the onset of APL are linked to a poor prognosis. If an APL case has additional chromosomal abnormalities thereafter during the disease progression, we would apply the standard treatment for APL, including all-trans retinoic acid (ATRA) and arsenic oxide (ATO), agents that result in better outcomes. Thus, because the diagnosis is critical to the treatment plan, it would be prudent to determine whether the APL has evolved from MDS (MDS/APL) and whether the APL occurred with additional chromosomal abnormalities.

We encountered an APL patient complicated with some clonal cytogenetic abnormalities including del(5q) and -6.

## Case presentation

A 30-year-old Japanese female presented with pancytopenia that had gradually progressed over 6 months prior to the onset. She was afebrile and did not have apparent abnormal physical examinations. She was referred to a hematologist for the examination of pancytopenia: her white blood cell count was 860/μL, hemoglobin 7.8 g/dL, and platelet 4.0 × 10^4^/μL. This was the so-called ‘preleukemic aplasia’ status. Coagulopathy was revealed by elevated FDP at 23.6 μg/mL (normal range, 0.0–5.0). Immediately after her visit to our outpatient hematology division clinic, we performed bone marrow aspiration, which revealed a high level of promyelocytes (70.0 %) in her bone marrow.

**Fig. 1:**
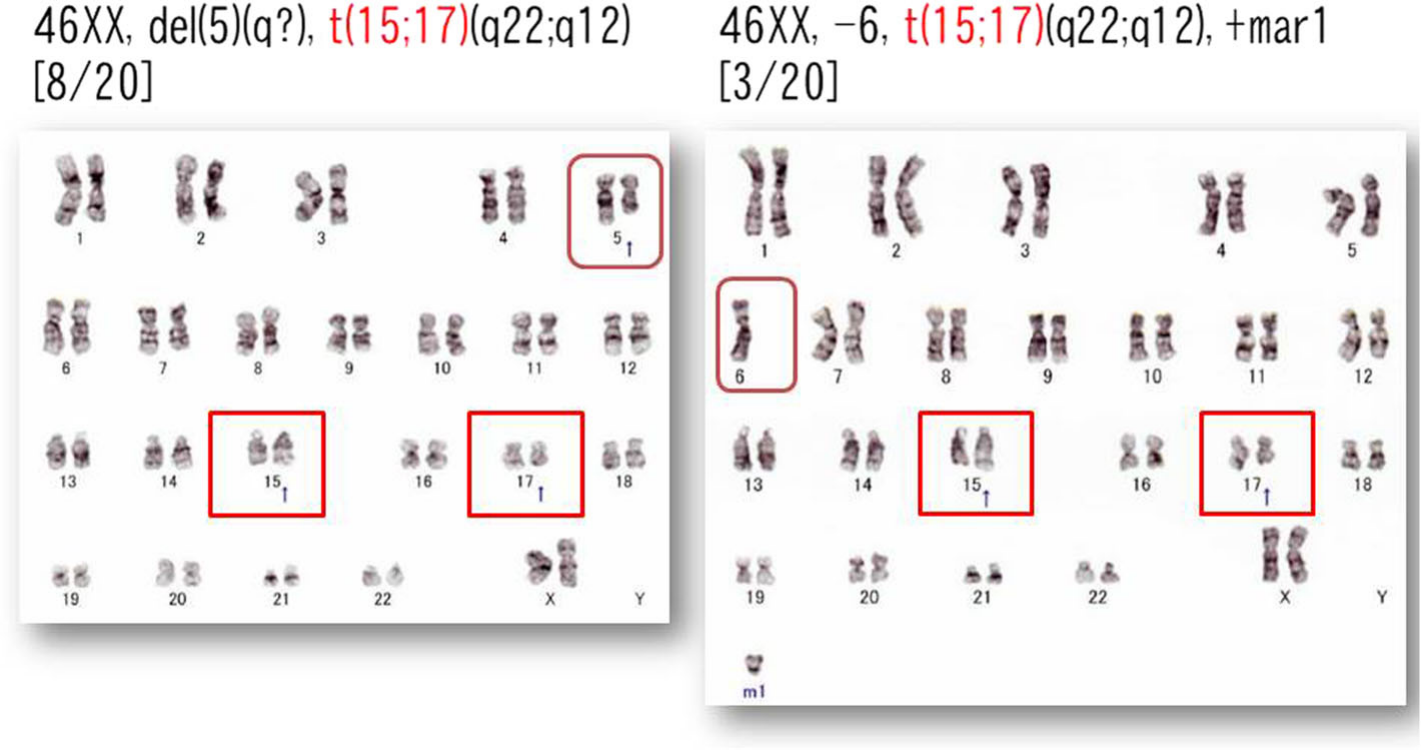
The chromosomal abnormality at the onset. Red squares indicate chromosomal abnormality t(15;17)(q22;q12), and brown rounded squares indicate del(5q) or −6 found simultaneously in each clone

A flow cytometry analysis showed that the aberrant myeloid cells expressed CD13^dim^, CD33+, CD117+, and lacked HLA-DR, CD34 and CD11b, leading to the diagnosis of APL, and thereafter the detection of *PML*-*RARA* fusion genes by a fluorescence *in situ* hybridization (FISH) analysis showed that 97 % of the analyzed cells bore the targeted fusion gene. This molecular detection confirmed the APL diagnosis after the initiation of induction chemotherapy consisting of ATRA [4]. A chromosomal analysis during the metaphase revealed that each of the tumor cells harbored del(5q) or −6 simultaneously with t(15;17)(q22;q12) (Fig. 1).

The ATRA therapy (45 mg/m^2^, until disease remission) resulted in the patient’s cytogenetic complete remission by 45 days after the induction of chemotherapy. The remission was indicated by the undetectable status of t(15;17) by chromosomal analysis. At 60 days after the initiation of ATRA chemotherapy, her cytopenia had recovered to the normal range. She switched over to an outpatient clinic on day 57. She achieved molecular remission proven by no PCR amplification of *PML*-*RARA* mRNA (<3.0 × 10^6^ copy/μg mRNA) at 16 weeks (day 112) with continuous ATRA treatment. She has maintained molecular remission as of 2 years.

## Conclusions

MDS is a hematological neoplasm that manifests as a few cytopenia lineage. Clonal cytogenetic aberrations (CCAs) are found in 30 %–50 % of primary MDS cases. AML evolving from MDS is a natural course of the disease, but the FAB classification M3 type conversion overt from MDS is a relatively rare form [3]. In our patient, antecedent MDS manifestations had been obscure clinically and morphologically. However, according to the WHO classification, all cases of AML harboring *PML*-*RARA* should be diagnosed as ‘AML with recurrent genetic abnormalities,’ even if the case has other chromosomal abnormalities. Moreover, the recurrent genetic abnormalities such as t(8;21)(q22;q22), inv(16)(p13.1q22), t(15;17)(q22;q12) and t(9;11)(p22;p23) are exclusively recognized as AML because of the genetic characteristics required for the leukemogenesis.

Regarding the concept of clonal expansion in hematological oncogenesis, it is conceivable that a common phenotypic or genetic feature occurs first [5, 6]. According to this logic, the clone with *PML*-*RARA* is original in the present case, and thereafter it obtained some additional chromosomal abnormalities such as del(5q) and −6. The concomitant appearance of *PML-RARA* and some of the recurring chromosomal abnormalities that characterize MDS is rare. The current understanding is that the *PML*-*RARA* fusion gene encoding a chimeric protein is required but not sufficient for leukemogenesis [7]. The molecular pathogenesis supports the hypothesis that *PML*-*RARA* rearrangement is one of the favorable molecular markers [8]. A truncated granulocyte-macrophage colony-stimulating factor (GM-CSF) allele on a 5q chromosome had been known in acute promyelocytic leukemia cell line, HL-60 [9]. This molecular pathology extends in a loss of other genes located on 5q chromosome, such as IL-3, IL-4, IL-5, and GM-CSF [10]. Those growth factor and cytokines are critical to differentiation within the lineage of the leukemic stem cell which carries del(5q) [10]. Then acute promyelocytic leukemia with del(5q) might have good prognosis compared to acute promyelocytic leukemia without del(5q). This should be determined in the clinical observation.

On the other hand, it is well recognized that therapy-related secondary hematological malignancies commonly occur after the remission of APL. The reported incidence of subsequent MDS or AML in patients treated for APL is 6.5 % [11–13]. However, the question of whether this oncogenesis is therapy-related or is derived from the genetic fragility of APL itself is under discussion from a molecular pathology point of view [14]. This fragility could contribute to the genetic fluctuation of APL [15]. In our patient, nevertheless it is a rare case, another possibility of explanation for AML etiology should be considered; AML evolved MDS, and one of the MDS clones kept containing 5q- molecular alternation. If so, the patients would be strongly recommend to undergo stem cell transplantation for the underlying MDS. In a while, it was reported that secondary APL is similar to de novo APL and should be considered distinct from other secondary acute myeloid neoplasms [16]. In our patient, ATRA treatment easily led to a complete molecular remission. Fortunately, 5-azacitidine and lenalidomide appear to be effective for such patients, but not all cases of APL with an additional chromosomal abnormality including variant APL are successfully treated with ATRA [17, 18].

We speculate that potential leukemogenesis could be anticipated in APL patients throughout their lives, presumably underlying the genetic instability of hematopoietic stem cells. Our review of all the rare cases cited herein [2, 17, 18] indicates that a further aggregation of APL cases with additional chromosomal abnormalities is needed for the determination of the clinical gravity, optimal treatment, and degree of background chromosomal risk based on the differences in genetic prognosis.

We propose that an assessment of additional CCA in APL would contribute to the clinical decisions regarding whether to treat APL with cytotoxic agents. It would be of interest to know the extent of CCA in the patients regarding to mixed leukemia. One or more additional cytogenetic abnormalities other than *PML-RARA* could account for the biological malignant grade and prognostic index.

## Abbreviations

APL: Acute promyelocytic leukemia
ATO: Arsenic oxide
ATRA: All-trans retinoic acid
CCA: Clonal cytogenetic aberration
FISH: Fluorescence *in situ* hybridization
MDS: Myelodysplastic syndrome
MDS/APL: APL evolved from MDS
